# Reversible Cortical Visual Impairment in an Adolescent Due to a Posterior Fossa Arachnoid Cyst: A Case Report

**DOI:** 10.3390/life15071121

**Published:** 2025-07-17

**Authors:** Jelena Škunca Herman, Dario Josip Živković, Ivana Orešković, Lana Knežević, Maja Malenica Ravlić, Blanka Doko Mandić, Goran Marić, Ante Vukojević, Hrvoje Sliepčević, Mia Zorić Geber, Vladimir Kalousek, Zoran Vatavuk

**Affiliations:** 1 Department of Ophthalmology, Sestre Milosrdnice University Hospital Centre, 10000 Zagreb, Croatia; jelena.skunca@kbcsm.hr (J.Š.H.); ivana.oreskovic@kbcsm.hr (I.O.); lana.knezevic@kbcsm.hr (L.K.); maja.malenica@kbcsm.hr (M.M.R.); blanka.doko@kbcsm.hr (B.D.M.); goran.maric@kbcsm.hr (G.M.); mia.zoric@kbcsm.hr (M.Z.G.); zoran.vatavuk@kbcsm.hr (Z.V.); 2 Department of Neurosurgery, Sestre Milosrdnice University Hospital Centre, 10000 Zagreb, Croatia; dario.josip.zivkovic@kbcsm.hr; 3 Department of Ophthalmology, General County Hospital Vinkovci, 32100 Vinkovci, Croatia; hrvoje.sliepcevic@gmail.com; 4 Department of Diagnostic and Interventional Radiology, Sestre Milosrdnice University Hospital Centre, 10000 Zagreb, Croatia; vladimir.kalousek@kbcsm.hr

**Keywords:** arachnoid cyst, cerebral compression, cortical visual impairment, cystoperitoneal shunt, neuroimaging, posterior fossa, primary visual cortex, reversible vision loss

## Abstract

**Background**: Arachnoid cysts are typically benign and asymptomatic, but large cysts can exert a mass effect on adjacent neural structures. Based on the available literature, no cases of cortical visual impairment (CVI) in an adolescent caused by posterior fossa arachnoid cysts have been reported. **Case presentation**: We report the case of a previously healthy 16-year-old girl who presented with sudden and rapidly progressive bilateral visual loss due to a large retrocerebellar arachnoid cyst. She reported blurred vision, tunnel vision-like, and decreased visual acuity. Although neuro-ophthalmologic and imaging workup revealed no damage to the anterior visual pathways, she exhibited progressive visual decline. Functional tests confirmed bilateral cortical visual impairment: pattern-reversal visual evoked potentials (VEPs) showed preserved and symmetric P100 latencies and amplitudes, while automated perimetry revealed bilateral concentric visual field constriction with preserved central islands. Following cystoperitoneal drainage, her vision rapidly and completely recovered. **Conclusions**: To the best of our knowledge, this is the first reported case of reversible CVI in an adolescent caused by a posterior fossa arachnoid cyst without intracranial pressure (ICP) elevation or optic nerve involvement, and with tunnel vision-like. Our findings emphasize the role of posterior fossa lesions in visual dysfunction and highlight the potential reversibility of cortical visual loss when timely decompression is achieved. This case underscores the importance of including posterior fossa lesions in the differential diagnosis of unexplained bilateral visual loss, even in the absence of elevated intracranial pressure or anterior visual pathway involvement.

## 1. Introduction

Arachnoid cysts, located within the arachnoid membrane, are non-malignant, cerebrospinal fluid-filled sacs that are often asymptomatic and found incidentally [1]. Prevalence ranges from approximately 1.4% to 2.6% depending on the population studied [2,3], and around 1–2% in the general population [4].

Most are congenital, though they may also be acquired following trauma, infection, or hemorrhage [3]. The most frequent location for arachnoid cysts is the middle cranial fossa. Other common sites include the posterior fossa and suprasellar region [2]. However, depending on their size and location, these cysts can sometimes cause neurological symptoms, commonly headaches, seizures, or signs of increased intracranial pressure (ICP), and even visual disturbances [4,5,6].

The exact prevalence of visual disturbances among individuals with arachnoid cysts is not well defined in the literature. Visual symptoms are relatively rare, reported in approximately 4% of cases in some surgical series [7]. When present, they typically result from compression of the optic nerves, chiasm, or visual pathways, particularly in suprasellar or middle cranial fossa cysts [8]. Documented visual disturbances include progressive visual loss, visual field defects and papilledema. However, visual impairment due to posterior fossa arachnoid cysts, although rare, may present as quandrantopia or hemianopia [9,10].

Differential diagnoses of posterior fossa cystic lesions include Dandy-Walker malformation, Blake pouch cyst, and mega cisterna magna. While these entities may present with overlapping radiologic features, they differ in embryological origin, clinical significance, and treatment implications [11,12].

Surgical options for symptomatic cysts typically include endoscopic fenestration, open microsurgical fenestration, or cystoperitoneal shunting, depending on the cyst’s location, size, and communication with surrounding cerebrospinal fluid (CSF) spaces [4].

This case report presents a unique instance of reversible cortical visual impairment (CVI) in an otherwise healthy adolescent. CVI is defined as visual dysfunction resulting from damage or dysfunction of the occipital lobes or posterior visual pathways, in the absence of primary ocular pathology [13].

It is more commonly described in pediatric patients with perinatal hypoxic-ischemic injury or neurodevelopment disorders, but may also occur due to compressive lesions [14,15]. CVI typically presents with bilateral visual loss, often with preserved pupillary reactions and normal ocular findings. Characteristic visual field defects may include homonymous hemianopia, inferior or superior quadrantopia, or concentric peripheral field constriction resembling tunnel vision-like [13,16].

These patterns reflect the involvement of specific regions within the visual cortex. CVI can be supported by functional tests such as visual evoked potentials (VEPs), which may show delayed or reduced responses, although findings can also be within normal limits depending on the extent and location of cortical dysfunction.

In our patient, this was caused by a giant retrocerebellar arachnoid cyst compressing the primary visual cortex. It underscores the importance of considering posterior fossa lesions in the differential diagnosis of unexplained bilateral visual loss.

## 2. Case Report

**Figure 1:**
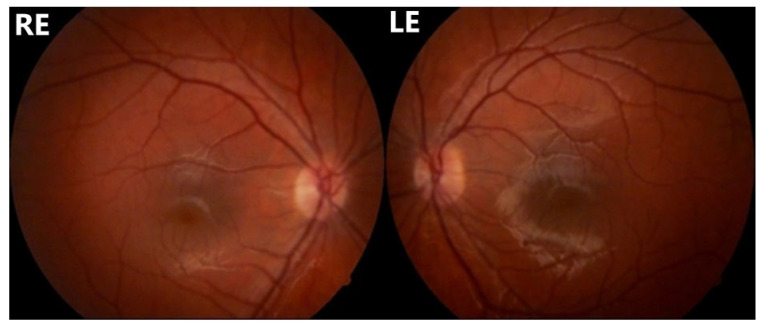
Funuds photography of both right and left eye showing no abnormalities.

**Figure 2:**
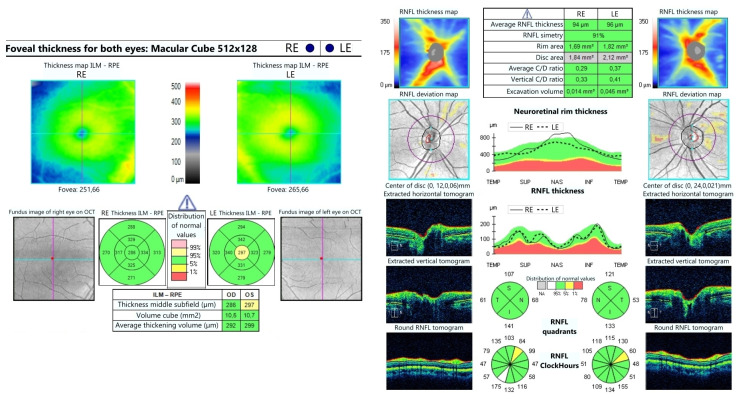
OCT scans of the macula and RNFL of both right and left eye showing no abnormalities.

**Figure 3:**
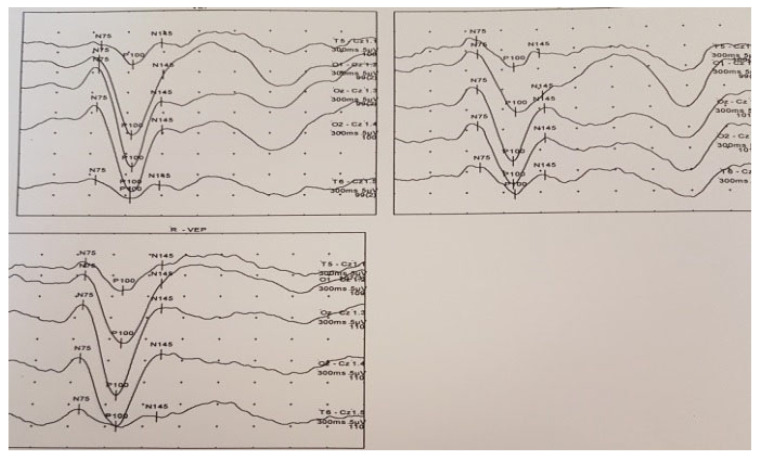
Visual evoked potentials (VEP) showing preserved P100 responses.

**Figure 4:**
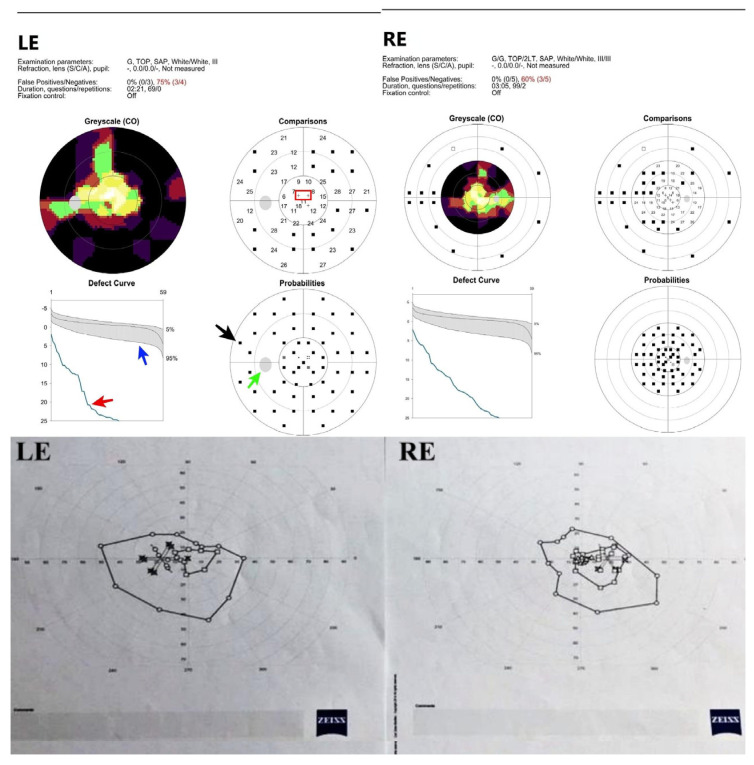
Preoperative visual field with bilateral peripheral loss. Upper panels show detailed functional visual field assessments, while the lower panels display global isopters for kinetic perimetry. Red arrow points to a section markedly below the 5th percentile of reference range (blue arrow), indicating a generalized depression of retinal sensitivity. The green arrow indicates the physiological blind spot. The black arrow marks a cluster of test points with significantly reduced sensitivity (*p* < 0.5%), representing a pathological paracentral scotoma not attributable to the blind spot. The red rectangle in the comparison plot encloses a location with a high degree of deviation from normative values, consistent with a localized and clinically relevant field defect.

**Figure 5:**
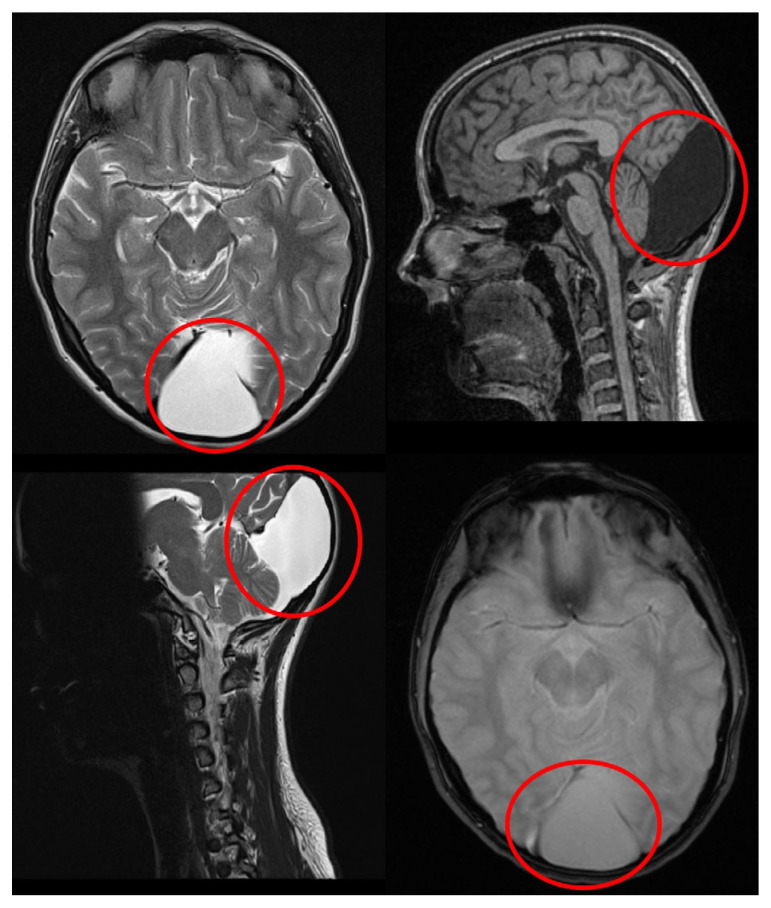
MRI revealing large retrocerebellar arachnoid cyst (red circles) compressing the cerebellar hemispheres and vermis, with upward displacement of the tentorium and cranial extension toward the occipital lobes. T2-weighted sequences revealed a large CSF-intensity cyst without communication with the fourth ventricle. No signs of hydrocephalus or optic pathway involvement were observed.

**Figure 6:**
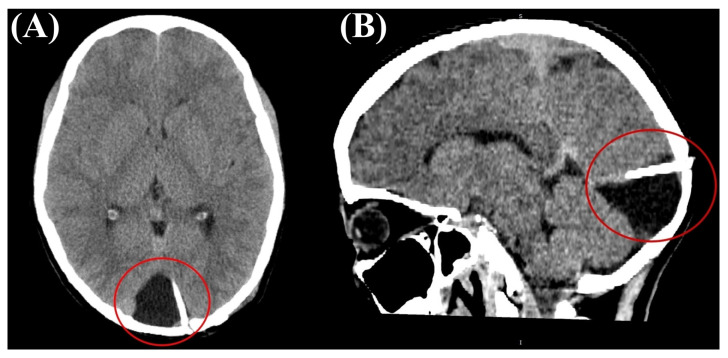
Postoperative computed tomography (CT) confirming the placement of the cystoperitoneal shunt (red circle). (**A**) Axial CT scan. (**B**) Sagittal CT scan.

**Figure 7:**
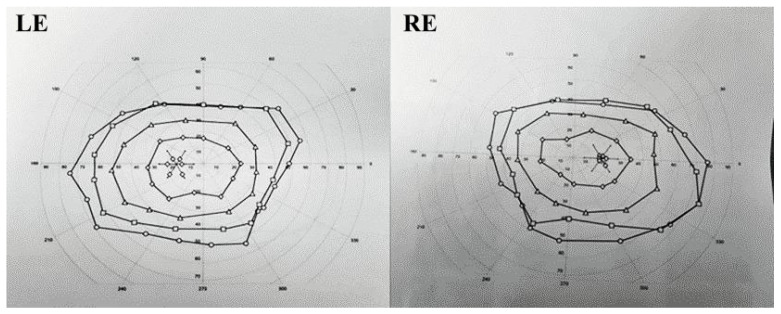
Postoperative visual field with expansion of isopters.

A 16-year-old girl, previously healthy, presented with sudden onset of visual disturbance, predominantly in the left eye. Symptoms began three days prior to presentation and progressively worsened, with the patient noting blurred vision, impaired color perception, and peripheral visual field defects. On examination, best corrected visual acuity (BCVA) was 0.0 logMAR in the right eye (RE) and 0.5 logMAR in the left eye (LE). Color vision assessed with Ishihara plates, was normal in the RE and mildly reduced in the LE, without a specific pattern of dyschromatopsia. Pupillary reflexes were symmetric and no relative afferent pupillary defect (RAPD) was detected. Anterior segment and fundus examination (Figure 1) were unremarkable in both eyes. Optical Coherence Tomography (OCT) of the macula and retinal nerve fiber layer (RNFL) revealed no abnormalities (Figure 2). Pattern-reversal visual evoked potentials (VEPs) showed preserved and symmetric P100 latencies and amplitudes bilaterally, within normal limits (Figure 3). Four days after initial presentation, BCVA further declined to 0.5 logMAR (RE) and 1.0 logMAR (LE). Visual field testing (Octopus and Humphrey kinetic perimetry) demonstrated bilateral concentric field constriction (“tunnel vision-like”) with preserved central islands (Figure 4). Given the discrepancy between profound visual loss and normal ocular and electrophysiological findings, a cortical etiology was suspected. Brain Magnetic Resonance Imaging (MRI) revealed a large retrocerebellar arachnoid cyst (approx. 4 × 4 × 6 cm) exerting mass effect on adjacent structures. There was displacement of the tentorium and compression of the cerebellar hemispheres and vermis, without signs of atrophy. The cranial extension of the cyst toward the occipital lobes suggests a mass effect on the primary visual cortex (V1). The cyst did not communicate with the fourth ventricle, Standard neuroimaging sequences (T1-T2, FLAIR, and T2 GRE) clearly delineated the arachnoid cyst without any visible solid component, surrounding edema, or evidence of additional cysts. Hemorrhage was excluded based on the absence of blooming artifact on T2 GRE sequences, and the lack of visible vasogenic edema ruled out a neoplasm. These imaging characteristics, together with the lesion’s extra-axial location and signal equivalence to CSF across all sequences, were consistent with a non-neoplastic arachnoid cyst. There were no additional intracranial abnormalities such as agenesis of the corpus callosum or hydrocephalus (Figure 5). The optic nerves and chiasm appeared morphologically and signal-wise normal. Due to the progressive visual symptoms and confirmed mass effect, the patient underwent neurosurgical intervention. A cystoperitoneal shunt was placed using a low-pressure Pudenz valve (Figure 6A). The procedure was uneventful. The proximal catheter was placed as cranially and laterally as possible within the cyst cavity, with the aim of avoiding flow obstruction and minimizing discomfort during lying. This placement was chosen due to the anatomical configuration of the cyst and was verified intraoperatively and via postoperative imaging. As shown in the MRI images, the upper portion of the cyst lies sufficiently cranially to avoid direct contact with hard surfaces when the patient is supine (Figure 6B). On postoperative day 1, BCVA improved to 0.3 (RE) and 0.5 (LE). At 40 days post-surgery, BCVA had normalized to 0.0 (RE) and 0.1 (LE), with significant improvement in visual fields. Postoperative kinetic perimetry showed clear expansion of isopters in all meridians (Figure 7). The patient had no other neurological symptoms or history of trauma, infection, or systemic illness. She remained stable at follow-up, with full visual recovery.

Parental consent for publication was obtained.

## 3. Discussion

To our knowledge, this is the first documented case of reversible cortical visual impairment (CVI) in an adolescent caused by a posterior fossa arachnoid cyst without signs of elevated intracranial pressure or optic nerve involvement. The patient presented with rapidly progressive bilateral visual loss despite normal findings on ophthalmologic, electrophysiological, and anterior visual pathway assessments. Neuroimaging revealed a large retrocerebellar arachnoid cyst compressing the occipital lobes, with imaging features consistent with a non-neoplastic, CSF-equivalent lesion, as shown in Figure 5.

In this context, our case illustrates the importance of recognizing arachnoid cysts as a potentially reversible cause of CVI, particularly in pediatric patients.

Although often asymptomatic, arachnoid cysts may produce visual symptoms through various mechanisms. In most reports, visual loss results from optic nerve or chiasmal compression in suprasellar or parasellar locations, or due to increased intracranial pressure (ICP) leading to papilledema and optic neuropathy [17,18]. However, our patient had no signs of optic disc swelling or hydrocephalus and had normal VEPs and OCT, ruled out anterior visual pathway dysfunction. The visual loss was thus attributed to direct mechanical compression of the occipital lobes by the cyst.

Surgical options were discussed in interdisciplinary consultation. Given the retrocerebellar location of the cyst and its smooth, non-lobulated morphology without septations, cystoperitoneal shunting was selected as the safest and least invasive option. Current literature supports shunting as a valid and widely accepted alternative when direct fenestration is technically challenging due to location or morphology [19]. Our approach was tailored to the patient’s anatomy and the need for urgent decompression, prioritizing visual recovery while minimizing operative risk. Complete visual recovery following neurosurgical decompression underscores the reversibility of CVI when recognized and managed promptly.

This case contributes to the limited literature on CVI caused by a posterior fossa arachnoid cyst. The patient developed progressive bilateral visual loss due to direct occipital compression, with complete recovery following neurosurgical decompression. Cortical involvement was confirmed by visual field testing and electrophysiology, despite normal ICP and optic nerves.

A similar case was reported by Suzuki et al., involving an elderly woman with a recurrent convexity occipital arachnoid cyst and partial quadrantanopia, but no functional confirmation of cortical involvement [9]. Additionally, Yagi et al. described a right occipital convexity arachnoid cyst in a 51-year-old woman with slowly progressive homonymous hemianopia which resolved after decompression [10]. In contrast, our adolescent patient presented acutely, tunnel vision-like, with bilateral CVI, confirmed by objective testing and fully resolved after shunting, highlighting key differences in age, cyst location, and outcome.

Other pathologies such as tumors, infarction, or hemorrhage in the occipital lobes may also present with reversible visual symptoms after treatment [20,21,22].

We hypothesize that the sudden visual loss may have resulted from recent cyst expansion, possibly due to altered CSF dynamics or a transient “one-way valve” mechanism, as previously proposed in the literature [9]. This may explain the sudden onset and rapid progression of visual symptoms in our patient. Furthermore, although tunnel vision is typically linked to retinal or optic nerve damage, the bilateral concentric field constriction with reduced acuity likely reflects cortical dysfunction due to occipital compression (tunnel vision-like) [23].

The mass effect of the cyst resulted in upward displacement of the tentorium and compression of the cerebellar hemispheres and vermis, as seen on MRI (Figure 5). Importantly, the cranial extension of the cyst brought it into close proximity with the occipital lobes, suggesting compression of the primary visual cortex (V1), which may explain the reversible CVI observed in our patient. Although no direct signs of raised ICP were observed, these structural changes may have contributed to transient cortical dysfunction through posterior fossa crowding and altered CSF dynamics. The lesion was well-defined, with CSF-equivalent signal on all sequences, and no communication with the fourth ventricle. There were no additional anomalies such as vermian dysgenesis or agenesis of the corpus callosum. These findings helped exclude other posterior fossa cystic entities such as Dandy-Walker malformation (typically associated with vermian dysgenesis and hydrocephalus), Blake pouch cyst (which communicates with the fourth ventricle), and mega cisterna magna (which lacks mass effect and structural displacement) [11,12]. These findings supported the diagnosis of a symptomatic, isolated arachnoid cyst requiring neurosurgical intervention.

This case highlights the importance of including CVI in the differential diagnosis of bilateral visual loss when neuroimaging reveals posterior fossa lesions with mass effect on the occipital cortex (V1). The anatomical proximity of the cerebellum to the visual cortex, particularly near the tentorium, renders the occipital lobes susceptible to compression by expanding cyst even in the absence of hydrocephalus [24]. In our patient, this configuration was clearly demonstrated by MRI, supporting a pathophysiological mechanism involving direct compression of the primary visual cortex (V1). We hypothesize that the observed transient CVI resulted from chronic, subclinical mass effect impairing neuronal excitability or local microcirculation without inducing irreversible damage. This is further supported by the rapid visual recovery following neurosurgical decompression, suggesting functional suppression rather than structural loss [25].

This case highlights the importance of considering posterior fossa lesions, including arachnoid cysts, in the differential diagnosis of unexplained bilateral visual loss, particularly when ophthalmologic and anterior visual pathway assessments are normal. Visual symptoms are rarely cortical in children and often go unrecognized, especially in the absence of classic signs such as papilledema [7]. Previous reports have mostly described visual loss related to suprasellar or middle cranial fossa cysts causing raised intracranial pressure (ICP) and optic neuropathy [17,18]. Weil, R.J. described a sellar/suprasellar arachnoid cyst causing rapid vison loss due to optic chiasm compression, with full recovery after urgent fenestration [8]. Similarly, Ishii, T. et al. reported an adult with a middle cranial fossa cyst and acute optic nerve compression, whose vision improved after surgery [26]. Stubgen J.P. described a 75-year-old woman with a suprasellar cyst and progressive homonymous hemianopia despite no signs of raised intracranial pressure. After endoscopic cyst fenestration, her visual field defect fully resolved. These cases show that even without increased ICP, cysts can compress visual pathways and cause reversible deficits [27]. Menon, R.K. and Wester, K.G. described acute visual loss from a temporal arachnoid cyst after minor head trauma while Shin, C.J. et al. reported a posterior fossa cyst causing hydrocephalus and optic nerve compression in a 39-year-old. Despite surgery, vision did not recover in one case, illustrating how cysts can also lead to permanent damage via ICP-related optic nerve compression [1,28]. Mayeda, M.S. et al. found papilledema in 57% of 35 children with ruptured arachnoid cysts, many with reduced visual acuity despite hydrocephalus treatment. These findings emphasize the need for close ophthalmologic follow-up to prevent irreversible visual loss [29]. It is also important to highlight the study by Gotz Wieckowska et al., which analyzed ophthalmological symptoms in children with posterior fossa arachnoid cysts. While most patients exhibited ocular disturbances such as strabismus, nystagmus, or abnormal head posture, none underwent formal visual field testing, and CVI was not demonstrated. Notably, even the two children without ocular signs had neurological abnormalities. In contrast, our patient presented with isolated bilateral visual loss without any neurological or ocular motor signs, and objective testing confirmed cortical involvement, supporting a distinct pathophysiological mechanism [6].

Despite the striking clinical course and favorable outcome in this case, several limitations should be acknowledged. First, as a single case report, it does not allow for generalization of findings. Second, advanced functional neuroimaging techniques such as functional MRI (fMRI), perfusion imaging, and diffusion tensor imaging (DTI) were not performed. These modalities could have provided more detailed insight into the functional status of the visual cortex and optic pathways before and after decompression, and helped to better elucidate the underlying pathophysiology of CVI. Unfortunately, they were not available at our institution. Third, while a postoperative CT scan demonstrated resolution of mass effect and appropriate shunt positioning, the absence of follow-up MRI limited a more detailed assessment of cortical recovery and tissue integrity. Fourth, long-term follow-up is still ongoing, and recurrence or delayed complications cannot be entirely excluded. Nevertheless, this case adds to the growing body of literature emphasizing the need for heightened suspicion of cortical involvement in similar presentations. Early neurosurgical consultation may be crucial for preserving visual function.

## 4. Conclusions

This case represents the first reported instance of reversible CVI caused by a posterior fossa arachnoid cyst in an adolescent compressing the occipital lobes with tunnel vision-like. It highlights the importance of considering cortical etiologies in patients with unexplained bilateral visual loss, particularly when ophthalmologic and anterior visual pathway evaluations are unremarkable. The rapid and complete visual recovery following cystoperitoneal shunting suggests that cortical dysfunction due to mass effect can be reversible following timely decompression. Our findings emphasize the need for timely neuroimaging and multidisciplinary evaluation in similar cases, and broaden the clinical spectrum of arachnoid cyst presentation to include potentially reversible cortical visual disturbances. This case highlights the importance of prompt neurosurgical intervention in achieving full visual recovery.

## Data Availability

The original data generated and analyzed for this study are included in the published article. Further inquiries can be directed to the corresponding author.

## Abbreviations

The following abbreviations are used in this manuscript:

CVI	cortical visual impairment
ICP	intracranial pressure
BCVA	best corrected visual acuity
RAPD	relative afferent pupillary defect
OCT	Optical Coherence Tomography
RNFL	retinal nerve fiber layer
VEPs	visual evoked potentials
MRI	Magnetic Resonance Imaging
CT	Computed Tomography
CSF	Cerebrospinal fluid
